# Oxidative Stress and Endoplasmic Reticulum Stress Are Involved in the Protective Effect of Alpha Lipoic Acid Against Heat Damage in Chicken Testes

**DOI:** 10.3390/ani10030384

**Published:** 2020-02-27

**Authors:** Yongjie Xiong, Qirun Yin, Jing Li, Shaojun He

**Affiliations:** 1 College of Animal Science, Anhui Science and Technology University, Fengyang 233100, China; xiongyj@ahstu.edu.cn (Y.X.); qryin1990@163.com (Q.Y.); jingli1919@126.com (J.L.); 2 Key Laboratory of the Quality and Safety Control for Pork of the Ministry of Agriculture, Anhui Science and Technology University, Fengyang 233100, China

**Keywords:** heat stress, oxidative stress, endoplasmic reticulum stress, alpha lipoic acid, testis

## Abstract

**Simple Summary:**

In male animals, heat stress causes injury to the testes, resulting in an increase in the number of deformed sperm, a reduction in testosterone production, and consequently, reduced reproductive performance. As an important antioxidant, alpha lipoic acid (ALA) has been reported to have a protective effect against testicular injury caused by various pathological factors. However, few studies have focused on the role of ALA in heat-induced testicular lesions. In this study, the effects of ALA on histopathological parameters, the activity of key antioxidant enzymes involved in oxidative stress, biomarkers of endoplasmic reticulum stress signaling in the testicular tissue, and testosterone levels in serum were evaluated in heat-stressed chickens. The results showed that ALA significantly alleviated heat stress-induced adverse effects by affecting the activities of antioxidant enzymes, the expression of endoplasmic reticulum stress-related apoptotic modulators, and the protein levels of steroidogenic genes in the testes of chickens exposed to heat stress. These results suggest that in chickens, ALA may be beneficial for ameliorating decreased reproductive performance caused by heat stress and this study provides the basis for the design of novel therapies for heat-induced testicular damage.

**Abstract:**

Heat stress (HS) causes testicular injury, resulting in decreased fertility. Alpha-lipoic acid (ALA) is a well-known antioxidant. The aim of this study was to investigate the protective effects of ALA on HS-induced testicular damage in chickens. Histological changes; biomarkers of oxidative stress, including glutathione peroxidase (GPx), superoxide dismutase (SOD), catalase (CAT), and malondialdehyde (MDA); markers of endoplasmic reticulum (ER) stress, including glucose-regulated protein 78 (GRP78) and CCAAT/enhancer binding protein homologous protein (CHOP); apoptosis-related modulators, including Bax, Bcl-2, and caspase 3, in testicular tissue and serum testosterone levels were evaluated in chickens under heat stress. Heat stress induces spermatogenic cell abnormalities in chicken testes. Compared to the HS group, the histomorphological abnormalities in testicular tissue were visibly ameliorated, with significant increases in the enzyme activities of GPx, SOD, and CAT, increased serum testosterone concentration, and decreased MDA levels in the ALA + HS group. Consistent with these results, compared with the HS group, the protein levels of GRP78, CHOP, caspase 3, and Bax were significantly decreased, whereas Bcl-2, StAR, and 3β-HSD protein levels were increased in the ALA + HS group. Collectively, these findings suggest that ALA significantly ameliorates the heat-induced histomorphological abnormalities in the testes and decreased testosterone production by potentiating the activities of anti-oxidative enzymes (GPx, SOD, and CAT), inhibiting ER stress-related apoptotic pathways (Bax, Bcl-2, and caspase 3), and increasing steroidogenic gene (StAR and 3β-HSD) expression in chickens.

## 1. Introduction

Heat stress (HS), as a non-specific environmental stress response, has a negative effect on reproduction efficiency and growth performance in livestock and poultry. It is well-known that chickens are more susceptible to HS than other species, due to their high feed conversion efficiency and housing density. Consequently, due to the intensification of global warming and the high expenses of aeration-cooling, the increasing economic loss caused by HS has become a serious issue for the poultry industry [1]. In male animals, HS reduces sperm production and increases the number of deformed sperm by damaging testicular tissue [2]. Previous studies have shown that the degree of testicular injury is positively correlated to the decrease in reproductive performance caused by HS in male animals [3]. Although heat-induced testicular injury has previously been investigated, additional studies are required to fully understand the mechanism by which HS impairs the normal physiological function of the testicles and to explore more effective and economical therapeutic strategies.

Oxidative stress, which results from an imbalance between oxidant and antioxidant systems, induces damage to cells and tissues in animals [4]. At the same time, it has been demonstrated that an imbalance in the oxidative state increases the expression levels of apoptosis-related genes and triggers a series of signaling pathways, including activation of endoplasmic reticulum (ER) stress signaling, resulting in pathological lesions [5]. The ER is a ubiquitous and important organelle, responsible for the folding, assembly, and maturation of newly synthesized proteins in eukaryotic cells. When the ER self-homeostatic state is disrupted by multifarious physiological or pathological factors, the protein-folding capacity of the ER is affected. The accumulation of unfolded or misfolded proteins in the ER lumen then triggers the ER stress response [6]. As showed in previous studies, ER stress is considered to be a protective response and it plays an important role in maintaining or restoring normal physiological function to cells during the early stages of stress or damage [7,8]. However, persistent or severe ER stress leads to apoptosis [9]. Moreover, existing evidence demonstrates that heat-induced oxidative stress and ER stress are responsible for various types of injury to cells, tissues, and organs in chickens and other species. These include apoptosis, immune imbalance in the intestines, and nephropathy [10,11,12,13]. A recent study reported the use of antioxidants to protect the testis against the adverse effects of HS [14]. Moreover, oxidative stress and ER stress have been shown to play important roles in testicular damage caused by hyperthermia [3,15].

Alpha lipoic acid (ALA), a lipophilic compound found in prokaryotic and eukaryotic cells, has been widely applied to treat reproductive diseases due to its antioxidative and anti-inflammatory actions [16]. Moreover, it has previously been shown that ALA has a protective role against the decreased growth performance and meat quality of broilers under heat stress [17]. Nevertheless, it is still unclear whether ALA can protect the testes against HS-induced injury and whether oxidative stress and ER stress are involved in this process in chickens.

Therefore, the aim of this study was to investigate the protective effects of ALA on HS-induced histomorphological changes in chicken testes and to determine the involvement of oxidative stress and ER stress in this process. A better understanding of the protective effects of ALA against HS will provide the basis for the design of novel therapies for HS-induced reproductive dysfunction in male chickens.

## 2. Material and Methods

### 2.1. Animal Care and Experimental Treatment

All protocols used in the current study were approved by the Animal Care and Use Committee of Anhui Science and Technology University, Fengyang, China (License number: AHSTUDKY2016002). ALA was purchased from Guangzhou Shengxuan Biotechnology Co. Ltd., Guangzhou, China. The R/S mixed form of ALA was used and mixed in the feed according to a prior report [18]. Since there was no technique available for separating the R form from the R/S mix, we did not consider the optical activity of ALA. The grower diet (22–42 days) (Table 1) was formulated to meet National Research Council (NRC, 1994)-recommended requirements for all nutrients and according to the procedure described in our prior report [19], whereas the ALA diet was prepared by using a basal diet supplemented with ALA (500 mg/kg diet) based on a previous report [20]. The experimental treatment was performed based on our previous study but with minor modifications [19]. Briefly, a total of 200 chickens (28 days old; Arbor Acres, Fengyang, China) were randomly assigned into four groups with 50 chickens in each group as follows: control (C), ALA treatment with (ALA), HS, and HS with ALA treatment (ALA + HS). Chickens in groups C and HS received a basal diet and chicks in the ALA and ALA + HS groups were supplied with the ALA diet (500 mg/kg diet), daily for 14 days. The chicks in the C and ALA groups were kept at 24 ± 1 °C and 45–55% relative humidity (RH), while those in the HS and ALA + HS groups were subjected to heat stress treatment at 32 ± 1 °C and 55–65% RH for 6 h/day from 10:00 to 16:00, from day 28 to day 42. All chickens received ad libitum food and water throughout the entire experimental period. All the chickens were sacrificed at day 42 and blood samples and testes were immediately collected. One testis was immersed in liquid nitrogen for subsequent analyses, while the other testis was used for histological assessment. Serum was obtained from the blood samples and was stored at −80 °C in an ultra-low temperature freezer.

### 2.2. Histological Sections of Testes

After immersion in formaldehyde, followed by dehydration in different concentrations of alcohol, testis tissue was embedded in liquid paraffin. Following solidification, paraffin-embedded tissue samples were cut into a series of 5 μm sections using a rotary microtome (RM2235, Leica Biosystems, Buffalo Grove, IL, USA). Hematoxylin and eosin staining was performed as previously described [21]. In brief, the sections were treated with xylene and ethanol, washed with water, and then stained with hematoxylin for 10 min. After washing with water and immersing in a hydrochloric acid-alcohol solution, the sections were washed again and stained with eosin for 50 s. Subsequently, the sections were decolorized in water for 3 min and dehydrated gradually in a series of different concentrations of alcohol. The sections were soaked in xylene until transparent and a neutral resin was then added. After drying at room temperature, the sections were viewed and photographed using a digital optical microscope system.

### 2.3. Analysis of Oxidative Stress Markers

After homogenizing in cold physiological saline solution, testis samples were centrifuged at 500 g for 15 min to obtain supernatants for the biochemical analysis of oxidative stress markers. As described in a previous study [22], glutathione peroxidase (GPx), superoxide dismutase (SOD), and catalase (CAT) were analyzed using commercial kits, according to the procedure provided by the manufacturer (GPx assay kit, A005-1-2; SOD assay kit, A001-3-2; CAT assay kit, A007-1-1; Nanjing Jiancheng Biological Engineering Research Institute Co. Ltd., Nanjing, China). Malondialdehdye (MDA) levels were measured using a colorimetric method (MDA assay kit, A003-1-2, Nanjing Jiancheng Biological Engineering Research Institute Co. Ltd.). The protein concentration of all samples was determined using the Bradford method [23].

### 2.4. Western Blotting Analysis

The extraction of total protein from chicken testes and the measurement of its concentration were performed using commercial protein extraction and detection kits (KGP250 for extraction of total protein; KGPBCA for measurement of protein concentration; Keygen Biotech, Jiangsu Keygen Biotechnology Co. Ltd., Nanjing, China), according to the manufacturer’s protocols. A total of 30 μg of protein was separated by 12% sodium dodecyl sulfate-polyacrylamide gel electrophoresis and then transferred onto polyvinylidene difluoride (PVDF) membranes using an electrophoretic blotting system (Bio-Rad, Hercules, CA, USA). The membranes were completely immersed in blocking buffer (P0252-100, Beyotime Biotechnology, Shanghai, China) for 90 min at room temperature and were then incubated with anti-glucose-regulated protein 78 (GRP78) (1:1,000; Proteintech, Wuhan, China), anti-CHOP (1:1000; Proteintech), anti-Bax (1:1,000; Proteintech), anti-Bcl-2 (1:1000; Proteintech), or anti-caspase 3 (1:1000; Proteintech) primary antibodies at 4 °C for 10 h. After incubation, the PVDF membranes were washed twice with TBST (Tris-buffered saline containing 0.1% Tween 20), and then incubated with HRP-conjugated secondary antibody (1:5000; Beyotime Biotechnology) for 1.5 h at 37 °C. After washing with TBST, the protein bands were detected using a FluorChem HD2 gel imaging and analysis system (ProteinSimple, Santa Clara, CA, USA). Densitometric analysis of the immunoreactive bands was performed using Quantity One software 4.6.2 (Bio-Rad Laboratories, Hercules, CA, USA).

### 2.5. Analysis of Serum Testosterone Levels

Serum testosterone levels were measured using a testosterone enzyme-linked immunosorbent assay kit (Beyotime Biotechnology, Shanghai, China), according to the manufacturer’s instructions. The minimum detectable concentration was 0.07 ng/mL and the intra-assay and inter-assay coefficients of variation were <10% and <15%, respectively.

### 2.6. Statistical Analysis

The statistical analysis was performed based on a prior report with minor modifications [14]. Briefly, data are represented as the mean ± SE. SPSS statistical analysis software (version 17.0; SPSS Institute, Chicago, IL, USA) was used to analyze the differences between groups by a one-way ANOVA, followed by Dunnett’s t-test. Differences were considered significant at *p* < 0.05.

## 3. Results

### 3.1. Histological Observations in the Testicular Tissue of Chickens Exposed to Heat Stress

**Figure 1:**
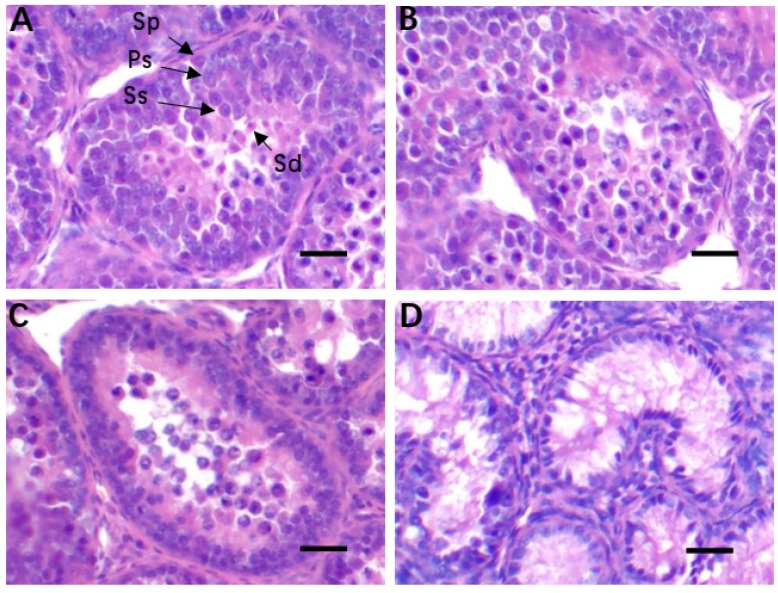
Observations of histological changes in testicular tissue of chickens exposed to heat stress (Hematoxylin and eosin staining). (**A**) Control group; (**B**) Alpha lipoic acid (ALA) treatment group; (**C**) Heat stress (HS) with ALA treatment (ALA + HS) group; (**D**) HS treatment group. Sp: spermatogonium; Ps: primary spermatocyte; Ss: secondary spermatocyte; Sd: spermatid. Scale bar 40 μm.

Numerous spermatogenic cell layers, with normal cell morphology were seen in the seminiferous tubules of testes from chickens in the control group (Figure 1A). However, abnormalities in the distribution of spermatogenic cell layers were observed in chickens of the HS group. Spermatocytes at different stages of development were rarely observed and spermatids were absent in the seminiferous tubules (Figure 1D). No remarkable differences were observed in the histological structure of testicular tissue in chickens from the ALA (Figure 1B) and control groups (Figure 1A). The changes in spermatogenic cell layers and structural abnormalities seen in the testis of chickens in the HS group (Figure 1D) were absent from those in the ALA + HS group (Figure 1C).

### 3.2. The Effect of Alpha Lipoic Acid on Oxidative Stress in the Testes of Chickens Exposed to Heat Stress

**Figure 2:**
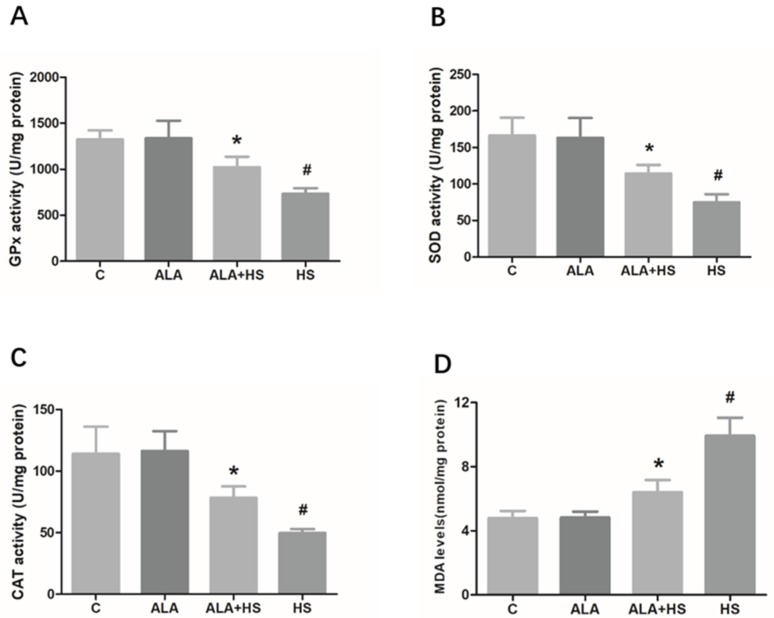
The effect of alpha lipoic acid on oxidative stress markers in the testes of chickens exposed to heat stress. Oxidative stress biomarkers, including (**A**) Glutathione peroxidase (GPx), (**B**) Superoxide dismutase (SOD), and (**C**) Catalase (CAT) enzyme activity, were analyzed using a colorimetric method with commercial kits. (**D**) Malondialdehyde (MDA) levels were measured using a colorimetric method. The statistical analysis results are shown as bar graphs. The data are presented as the mean ± SE of three independent experiments. Note: * *p* < 0.05 vs. HS group; **^#^** *p* < 0.05 vs. C group.

The levels of key biomarkers of oxidative stress, including GPx, SOD, CAT, and MDA, were analyzed using a commercial assay kit. HS treatment was found to reduce the activities of GPx (731.6 ± 35.90; *p* < 0.01), SOD (74.67 ± 6.489; *p* < 0.01), and CAT (49.67 ± 1.856; *p* < 0.01) (Figure 2A–C) and significantly increase the levels of MDA (9.933 ± 0.6467; *p* < 0.01) (Figure 2D). However, compared to the HS group, testicular GPx (1023 ± 64.67; *p* < 0.01), SOD (114.3 ± 6.766; *p* < 0.01), and CAT (78.33 ± 5.364; *p* < 0.01) activity were significantly increased, whereas MDA (6.403 ± 0.4419; *p* < 0.01) levels were significantly decreased in the ALA + HS group (Figure 2A–D).

### 3.3. The Effect of Alpha Lipoic Acid on Er Stress in the Testes of Chicken Exposed to Heat Stress

**Figure 3:**
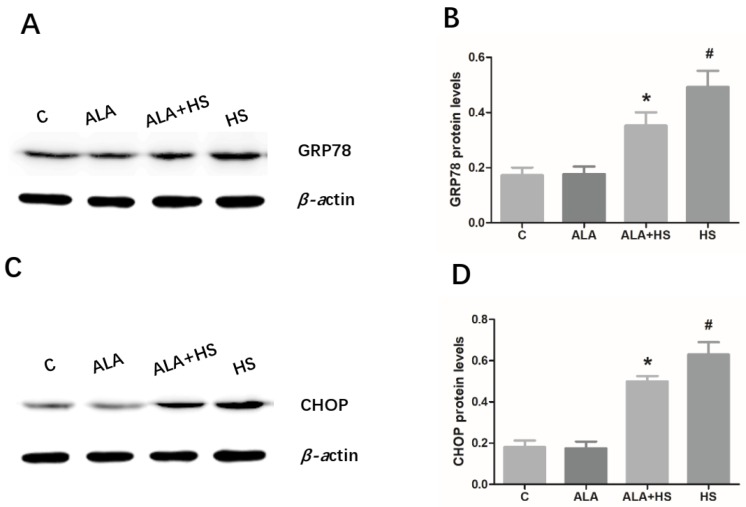
The effect of alpha lipoic acid on endoplasmic reticulum (ER) stress markers in the testes of chickens exposed to heat stress. Western blotting analysis of the expression of ER stress markers, (**A**) glucose-regulated protein 78 (GRP78) and (**C**) CCAAT/enhancer binding protein homologous protein (CHOP). (**B**) GRP78 and (**D**) CHOP protein expression levels were normalized to β-actin levels. The statistical analysis results are shown as bar graphs. The data are presented as the mean ± SE of three independent experiments. Note: * *p* < 0.05 vs. HS group; **^#^** *p* < 0.05 vs. C group.

The protein expression levels of the ER stress markers, GRP78 and CHOP, in chicken testes were analyzed by Western blotting. Similar to the results of oxidative stress analysis, heat treatment significantly increased the protein levels of GRP78 (0.4921 ± 0.03393; *p* < 0.01) and CHOP (0.6300 ± 0.03449; *p* < 0.01) (Figure 3A–D). However, the GRP78 (0.3522 ± 0.02776; *p* < 0.01) and CHOP (0.4998 ± 0.01477; *p* < 0.01) protein levels in chicken testes were significantly lower in the ALA + HS group than in the HS group (Figure 3A–D).

### 3.4. The Effect of Alpha Lipoic Acid on the Expression of Key Apoptosis-Related Modulators in the Testes of Chickens Exposed to Heat Stress

**Figure 4:**
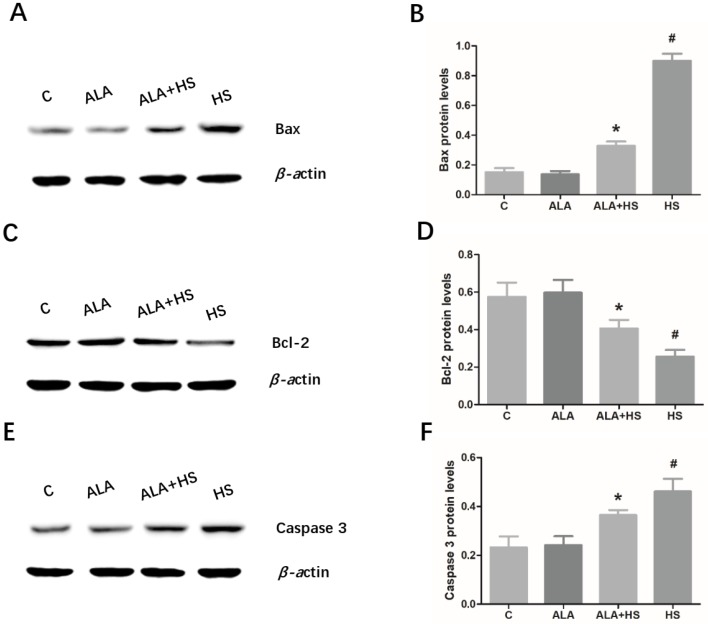
The effect of alpha lipoic acid on the expression of key apoptosis-related modulators in the testes of chickens exposed to heat stress. Western blotting analysis of the expression of apoptosis-related proteins, (**A**) Bax, (**C**) Bcl-2, and (**E**) caspase 3. (**B**) Bax, (**D**) Bcl-2, and (**F**) caspase 3 protein expression levels were normalized to β-actin levels. The statistical analysis results are shown as bar graphs. The data are presented as the mean ± SE of three independent experiments. Note: * *p* < 0.05 vs. HS group; **^#^** *p* < 0.05 vs. C group.

The expression levels of the key anti-apoptosis factor, Bcl-2 and the pro-apoptosis factors, Bax and caspase 3, were examined by Western blotting. The results indicated that heat treatment significantly increased Bax (0.9008 ± 0.02720; *p* < 0.01) and caspase 3 (0.4606 ± 0.03020; *p* < 0.01) protein expression levels (Figure 4A,B,E,F), but decreased Bcl-2 (0.2555 ± 0.02090; *p* < 0.01) expression levels (Figure 4C,D), suggesting that heat stress induced the activation of apoptosis in chicken testes. Compared to the HS group, the testicular expression levels of Bax (0.3284 ± 0.01662; *p* < 0.01) and caspase 3 (0.3641 ± 0.01193; *p* < 0.01) were significantly decreased and the expression level of Bcl-2 (0.4057 ± 0.02667; *p* < 0.01) was increased in the ALA + HS group (Figure 4A–F).

### 3.5. The Effect of Alpha Lipoic Acid on Serum Testosterone Levels in Chickens Exposed to Heat Stress

**Figure 5:**
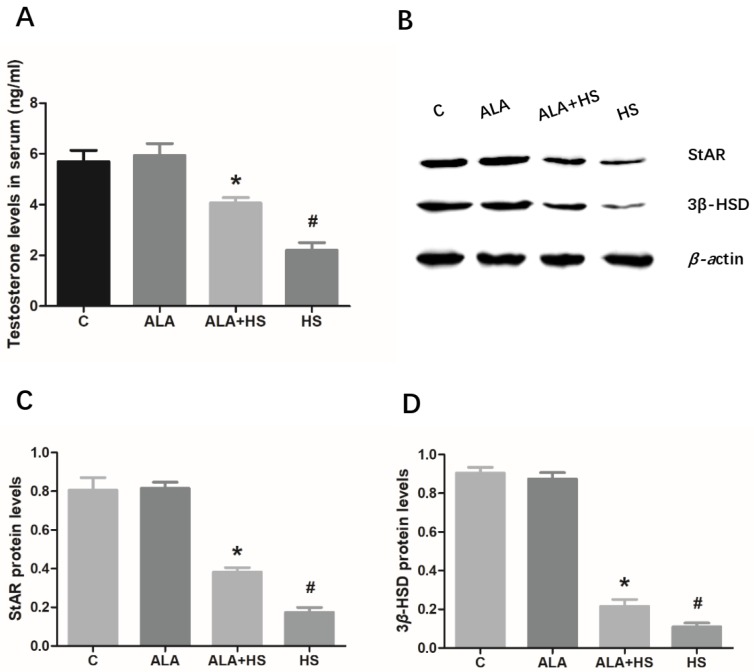
The effect of alpha lipoic acid on serum testosterone levels in chickens exposed to heat stress. (**A**) Serum testosterone concentration was determined using a commercial ELISA kit. (**B**) Western blotting was used to analyze the expression of the key steroidogenic-related proteins, StAR and 3β-HSD. (**C**) StAR and (**D**) 3β-HSD protein expression levels were normalized to β-actin levels. The statistical analysis results are shown as bar graphs. The data are presented as the mean ± SE of three independent experiments. Note: * *p* < 0.05 vs. HS group; **^#^** *p* < 0.05 vs. C group.

As shown in Figure 5A, compared to the control group, the concentration of testosterone (2.200 ± 0.1732; *p* < 0.01) was significantly decreased in the serum of heat stress-treated chickens, whereas ALA treatment significantly inhibited this effect. Moreover, ALA treatment led to an increase in the protein expression levels of StAR (0.3822 ± 0.01360; *p* < 0.01) and 3β-HSD (0.2164 ± 0.02026; *p* < 0.01), which are two key steroidogenic-related proteins involved in testosterone synthesis (Figure 5B–D).

## 4. Discussion

The pharmacologic activity of ALA has been widely studied due to its important antioxidant function in animals and humans. In rats, ALA has been found to attenuate testicular injury induced by exposure to various toxins by inhibiting oxidative stress in the testes [24,25]. In the current study, HS was found to cause histological changes in testicular tissue, as evidenced by hematoxylin and eosin staining. This may be one of the reasons for the decrease in reproductive performance seen in male animals under HS. Furthermore, it was found that the activity of antioxidant enzymes, including GPx, SOD, and CAT was significantly decreased, while MDA levels were increased in the testicular tissue of heat-stressed chickens, suggesting that a certain degree of lipid peroxidation and oxidative stress occurred in the chicken testicular tissue. MDA, which is one of the most important products of lipid peroxidation of the cell membrane and lipoproteins, has been shown to further aggravate lipid peroxidation, disrupting the process of mitochondrial electron transport and hence, interrupting the physiological function of mitochondria and affecting signal transmission pathways [26]. In living organisms, excessive reactive oxygen species (ROS) production following the oxidation of membrane lipids, induces MDA production. Moreover, MDA levels are often used to determine the degree of membrane lipid peroxidation [27]. In other words, MDA content is positively correlated with the degree of peroxidation-induced membrane damage. Usually, HS induces an acute increase in the levels of ROS, which are considered to be strong oxidizers [28]. Meanwhile, antioxidant enzymes, the most important defenders that significantly reduce the toxicity of ROS, have a protective effect against the adverse effects of lipid peroxidation [29]. Nevertheless, antioxidant enzymes, including GPx, SOD, and CAT, cannot effectively eliminate the increased amount of ROS due to persistent or severe HS, which induces an imbalance in redox status in vivo, resulting in excessive accumulation of MDA and oxidative damage to the tissue [30,31]. In this study, histological analysis of testicular tissue sections showed that HS caused abnormalities in the spermatogenic cell layers in seminiferous tubules, whereas these abnormalities were rarely observed in the ALA + HS treatment group. Furthermore, we found that ALA significantly enhanced GPx, SOD, and CAT activity, whereas it inhibited MDA production under heat stress. This may partly explain the anti-oxidative action of ALA in vivo reported in previous studies [32,33]. Based on these results, we speculated that HS caused a decrease in anti-oxidative capacity and disrupted the balance between oxidative and antioxidative status. However, ALA was found to restore this balance, indicating that it may have a protective role against hyperthermia-induced oxidative stress in chicken testes.

It is well established that several pathological signal transduction pathways, including ER stress and cell death, are triggered by oxidative stress [34]. ER stress, as a signaling pathway closely related to the regulation of apoptosis, may be one of the early responses of cells after damage or stress [35]. Existing evidence shows that ER stress plays an important role in physiological and pathological processes, such as the regulation of apoptosis and oxidative damage of cells and tissues [36,37]. Several studies have suggested that ER stress is triggered by HS and this process involves oxidative stress-related injury [38]. Sui et al. reported that oxidative damage is observed in mouse testes after exposure to HS [14]. Oxidative and ER stress also play important roles in bovine granulosa cells after HS [13]. Additionally, it has been shown that ALA inhibits oxidative lesions caused by methotrexate in rat testes [25] and it has a protective role in response to various cell and tissue injury processes, by inhibiting ER stress in vitro and in vivo [39,40]. Similar to these reports, the current study showed that HS induced higher protein expression levels of GRP78 and CHOP, two well-known ER stress markers, implying that ER stress was activated in chicken testes after exposure to HS. However, ALA treatment significantly reduced GRP78 and CHOP protein levels in the testes under conditions of HS. Thus, it can be inferred that ALA inhibited ER stress in the testes of heat-stressed chickens.

In addition, we detected changes in the protein expression of ER stress-related apoptosis modulators, including Bcl-2, Bax, and caspase 3. Previous studies have shown that CHOP, induced by ER stress, can down-regulate Bcl-2 expression levels and thus, induce apoptosis [41,42]. As important members of the Bcl-2 family, Bcl-2 has an anti-apoptotic effect, whereas Bax has a pro-apoptotic effect [43]. Moreover, caspase 3 is considered to be an executioner of apoptosis [44]. In the present study, HS induced up-regulated expression levels of Bax and caspase 3, but down-regulated Bcl-2 protein levels. However, in heat-stress conditions, ALA significantly inhibited the increase in Bax and caspase 3 protein levels and promoted Bcl-2 protein expression. This is consistent with previous reports that ALA protects cells and tissues against oxidative damage and cell death, such as renal tubular epithelial cell injury in humans [45] and oxidative damage of human retinal pigment epithelial cells [46], by regulating the expression of these ER stress-related apoptosis genes. Therefore, we infer that the down-regulation of Bcl-2 and the up-regulation of Bax and caspase 3 may be the mechanism whereby ALA ameliorated heat stress-induced histological changes and oxidative stress in chicken testes.

Testosterone is a steroid hormone mainly produced in the testes. It is well-known as an important indicator of the physiological function of the testes and it also plays an essential role in maintaining reproductive function in males, including spermatogenesis and maturation. A previous study has suggested that heat stress-induced oxidative stress results in decreased blood testosterone levels in mice [47]. Moreover, hyperthermia-induced ER stress is closely related to the decrease in testosterone production in mice exposed to HS [15]. To further confirm the protective role of ALA in ameliorating heat-induced testicular impairment, we investigated the effects of ALA on serum testosterone levels in chickens exposed to a high temperature. As expected, the decrease in serum testosterone concentration induced by heat stress was significantly restored by ALA treatment. Furthermore, the protein expression levels of StAR and 3β-HSD, two key enzymes that limit the rate of testosterone synthesis from the catalysis of cholesterol, were found to be up-regulated in the testis tissue of chickens after heat treatment. This implies that ALA has a protective effect against heat-induced testicular dysfunction, by increasing the expression levels of these steroidogenic genes, and thus increasing testosterone production by the testes.

In the past decade, there has been a growing number of investigations focusing on the effect of ALA supplementation on the antioxidant defense system in chickens. In female Recessive White Rock chickens (twenty-one-days-old), ALA (500 mg/kg diet) has been found to increase plasma SOD, GPx enzyme activities, and decrease plasma MDA activity [20]. Moreover, it has been demonstrated that dietary supplementation with 300 mg/kg ALA enhances the antioxidant capability and inhibits oxidative stress in liver and muscle in Ross male broiler chicks [48]. Previous reports have shown that ALA is an antioxidant because of its ability to scavenge free radicals, and two optical isomers of ALA seem to exist (R/S) [18,48]. However, little attention has been paid to the optical activity in these previous reports. In this study, we found that ALA (the R/S mixed form) significantly enhanced GPx, SOD, and CAT activity, and inhibited MDA production in testes from Arbor Acres chickens under HS, whereas there was no significant effect of ALA on these biomarkers of oxidative stress under normal farming conditions. However, it is worth noting that the composition and nutrient levels of basal diets, and chicken breeds were different in the aforementioned studies. To sum up, the protective action of ALA against oxidative stress in different tissues from chickens, and the related molecular mechanisms are not fully understood and need further investigation.

## 5. Conclusions

In summary, HS disrupted testicular tissue structure and induced oxidative stress and ER stress in chicken testes. Moreover, ALA ameliorated these detrimental effects of HS, possibly by enhancing the activity of antioxidant enzymes, inhibiting the ER stress-related apoptotic pathway, and up-regulating the expression of steroidogenic genes in chicken testes. These results suggest that dietary supplementation with 500 mg/kg ALA may alleviate heat-induced testicular lesions and improve reproductive performance in chickens under HS.

## Figures and Tables

**Table 1:** Composition and nutrient levels of basal diets (air-dry basis; %).

Item	22–42 d
Ingredients	
Maize	64.0
Soybean meal	27.8
Soybean oil	3.0
Fish power	2.5
Calcium hydrogen phosphate	1.4
Sodium chloride	0.30
Premix ^1^	1.0
Nutrient level	
ME/(MJ/kg) ^2^	13.03
Crude protein	20.02
Lysine	1.02
Methionine + Cystine	0.78
Calcium	1.00
Total phosphorus	0.67

^1^ The premix provided the following nutrients per kg of diets: Mn (as manganese sulfate) 66 mg, Zn 44 mg, Cu (as copper sulfate) 9 mg, Fe (as ferrous sulfate) 50 mg, I (as potassium iodide) 0.4 mg, vitamin A 7000 IU, vitamin D_3_ 875 IU, vitamin E 20 IU, vitamin K_3_ 1 mg, vitamin B_1_ 2 mg, vitamin B_2_ 4.5 mg, D-pantothenic acid 12 mg, nicotinic acid 50 mg, vitamin B_6_ 2.5 mg, vitamin B_12_ 0.6 mg. ^2^ Metabolizable energy (ME) was a calculated value. On the base of apparent metabolizable energy, the equation calculating ME can be expressed as ME = 13.7* maize% + 10.4* soybean meal% + 35.1* soybean oil% + 12.8* fish powder%.
